# *In vivo* evidence for the involvement of the carboxy terminal domain in assembling connexin 36 at the electrical synapse

**DOI:** 10.1016/j.mcn.2010.05.008

**Published:** 2010-09

**Authors:** Ingo Helbig, Esther Sammler, Marina Eliava, Alexey P. Bolshakov, Andrei Rozov, Roberto Bruzzone, Hannah Monyer, Sheriar Gustad Hormuzdi

**Affiliations:** a Department of Neuropediatrics, University Medical Center Schleswig-Holstein (UKSH), Schwanenweg 20, 24105 Kiel, Germany; b Department of Clinical Neurobiology, University of Heidelberg, Im Neuenheimer Feld 364, 69120 Heidelberg, Germany; c HKU-Pasteur Research Centre, 1/F, Dexter HC Man Building, Pokfulam, Hong Kong; d Centre for Neuroscience, Ninewells Hospital and Medical School, University of Dundee, Dundee, DD1 9SY, UK

**Keywords:** Connexin 36, Electrical synapse, Gap junction, Assembly, Transgenic, Intercellular channel, ZO-1

## Abstract

Connexin 36 (Cx36)-containing electrical synapses contribute to the timing and amplitude of neural responses in many brain regions. A Cx36-EGFP transgenic was previously generated to facilitate their identification and study. In this study we demonstrate that electrical coupling is normal in transgenic mice expressing Cx36 from the genomic locus and suggest that fluorescent puncta present in brain tissue represent distributed electrical synapses. These qualities emphasize the usefulness of the Cx36-EGFP reporter as a tool for the detailed anatomical characterization of electrical synapses in fixed and living tissue. However, though the fusion protein is able to form gap junctions between *Xenopus laevis* oocytes it is unable to restore electrical coupling to interneurons in the Cx36-deficient mouse. Further experiments in transgenic tissue and non-neural cell lines reveal impaired transport to the plasma membrane as the possible cause. By analyzing the functional deficits exhibited by the fusion protein *in vivo* and *in vitro*, we identify a motif within Cx36 that may interact with other trafficking or scaffold proteins and thereby be responsible for its incorporation into electrical synapses.

## Introduction

Connexin 36 (Cx36)-containing electrical synapses are abundant in the rodent brain wherein they facilitate sub- and supra-threshold synchrony and modulate or generate oscillations and seizure-like activity (Blatow et al., 2003; Buhl et al., 2003; Butovas et al., 2006; Deans et al., 2001; Hormuzdi et al., 2001; Landisman et al., 2002; Long et al., 2005; Pais et al., 2002). The functional consequences of these properties of electrical synapses are still incompletely determined, but recent reports of abnormal circadian activity, deficits in motor-coordination and motor learning, and impaired memory recall (Frisch et al., 2005; Long et al., 2005; Van Der Giessen et al., 2008) in Cx36-deficient mice demonstrate that transmission through electrical synapses is important for neuron and brain function.

Studies of gap junction coupling between interneurons in the cortex, amygdala, and hippocampus, shown to be Cx36-mediated in some instances, indicate that Cx36-containing electrical synapses show considerable specificity in their distribution. They demonstrate that an electrically-coupled neural circuit is primarily composed of neurons with a similar electrophysiological, immunocytochemical, and anatomical profile (Blatow et al., 2003; Caputi et al., 2009; Chu et al., 2003; Deans et al., 2001; Price et al., 2005; Woodruff and Sah, 2007). While exceptions exist (Caputi et al., 2009; Simon et al., 2005; Zsiros and Maccaferri, 2005), the above conclusion highlights the difficulty in studying electrical synapses by electrophysiological means since Cx36 gap junction-containing neurons may nevertheless be unresponsive in paired-cell recordings designed to detect gap junctions if the recorded cells belong to dissimilar interneuron subtypes. Electrophysiology in combination with methods to visualize gap junctions in the living slice may thus be best suited to study both, their prevalence and function. The findings also imply the existence of cell-specific functional or structural gap junction sorting mechanisms that create homocellular electrically-coupled circuits (Hormuzdi et al., 2004). Although speculative, recent reports that electrical synapse activity can be modulated (Arumugam et al., 2005; Kothmann et al., 2009; Landisman and Connors, 2005; Zsiros and Maccaferri, 2008) and that Cx36 interacts with the scaffold protein zonula occludens 1 (ZO-1) (Ciolofan et al., 2007; Flores et al., 2008; Li et al., 2004a; Rash et al., 2004) provide support for the existence of such regulatory mechanisms.

We previously described the generation of Cx36-EGFP, a transgenic line in which the Cx36 promoter in a Bacterial Artificial Chromosome derived from the mouse Cx36 locus regulates expression of an enhanced green fluorescent protein (EGFP)-tagged Cx36 variant (Christie et al., 2005; Feigenspan et al., 2004; Schubert et al., 2005). The expression of the fusion protein enabled the identification of Cx36-expressing cells and of the subcellular locations of gap junctions composed of the protein. In this study we show that the presence of distributed GFP puncta in the transgenic requires expression of wildtype Cx36 from the genome. Further electrophysiological characterization of the transgene and additional experiments in gap junction-deficient HeLa cells support a critical role for the carboxy terminal residues in assembling Cx36 into intercellular channels. Our observations provide important information on the assembly of Cx36-containing electrical synapses *in vivo* and, unexpectedly, highlight a unique property of the Cx36-EGFP transgenic reinforcing its use as an animal model to analyze the locations of electrical synapses composed of Cx36 in physiological and pathological conditions.

## Results

### Cx36-EGFP protein is a component of gap junctions in the transgenic cerebellum and olfactory bulb

**Fig. 1:**
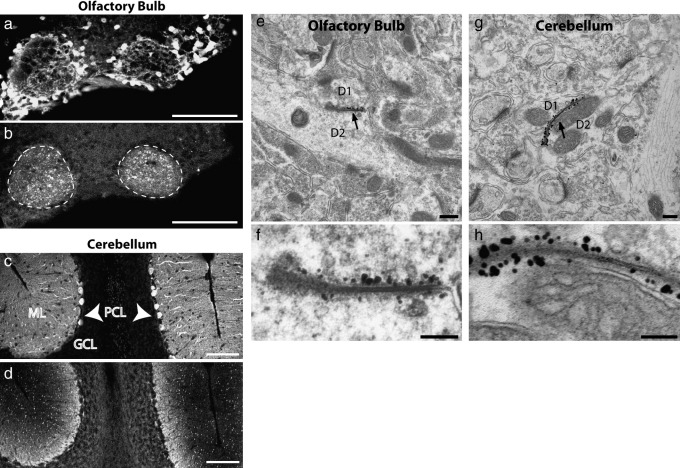
Cx36-EGFP protein is assembled into intercellular channels at the electrical synapse. GFP immunoreactivity (b,d) in combination with antibodies for calretinin (a) and calbindin (c) highlights the prominent distribution of Cx36-EGFP puncta within olfactory bulb glomeruli (b, dashed ovals) and in the molecular layer (ML) of the cerebellum (d, PCL = Purkinje cell layer indicated by arrowheads, GCL = granule cell layer). Immunoelectron microscopy with anti-GFP antibodies revealed the presence of EGFP-containing molecules at gap junctions (indicated by arrows; magnified f,h) between dendrites (D1 and D2) in the olfactory bulb (e,f) and cerebellum (g,h). Scale: a,b,c,d = 100 μM; e,g = 200 nM; f,h = 100 nM.

**Fig. 2:**
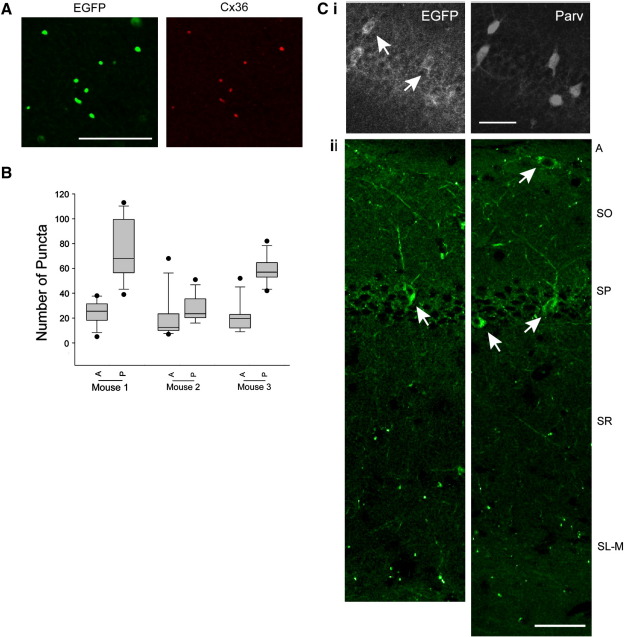
Cx36-EGFP puncta reflect the prevalence and distribution of Cx36-containing electrical synapses. A. A section of the Cx36-EGFP transgenic cerebellum demonstrating the colocalization of Cx36 puncta (red), detected with an anti-Cx36 antibody, with Cx36-EGFP fluorescent clusters (green). Only a small number of Cx36 immunopositive puncta were found to not colocalize with Cx36-EGFP fluorescent clusters. B. Measurement of Cx36-EGFP puncta quantity in the anterior and posterior (A and P respectively) striatum from 3 mice demonstrates a 2.4-fold average increase in prevalence in the latter. C.i. A portion of the transgenic hippocampus subjected to immunohistochemistry with anti-parvalbumin antibody (parv) illustrates the numerous parvalbumin-positive interneurons in the CA1 stratum pyramidale (SP) that also express the transgene (EGFP, arrows indicate examples of coexpressing interneurons). ii. Two images derived from the transgenic CA1 hippocampal region containing Cx36-EGFP-expressing interneurons (arrows) demonstrate the specific concentration of fluorescent puncta in the stratum oriens (SO) towards the alveus (A), and in the stratum radiatum (SR) near the stratum lacunosum-moleculare (SL-M). Scale: A = 25 μM, C = 50 μM.

**Fig. 3:**
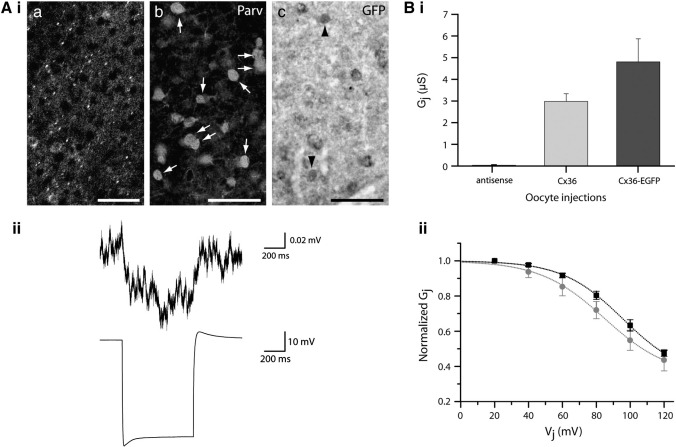
Cx36 function is retained after addition of the EGFP molecule to the carboxy-terminus of the protein. A. Immunohistochemistry of the neocortex with anti-GFP (ia) or with anti-parvalbumin (Parv) and anti-GFP (GFP) antibodies (ib,c) demonstrating the presence of puncta and of coexpression of Cx36-EGFP and parvalbumin indicating that parvalbuminergic interneurons in the neocortex express the transgene (arrows 1b; arrowheads in 1c demonstrate the presence of the fusion protein in parvalbumin-negative neurons). Electrophysiological experiments indicated that the transgene is unable to provide electrical coupling to FS neurons in the Cx36 knockout. Representative traces (postsynaptic response above, presynaptic below) obtained from a pair exhibiting a CC of 0.0014 demonstrate our ability to detect low levels of gap junction-mediated communication in such experiments (ii). B. The junctional conductance (*G*_j_, mean ± SEM) measured from oocytes injected with RNAs encoding either of the two Cx36 variants was similar and significantly higher than those measured from oocytes injected solely with the oligonucleotide controls (Bi). The relationship of *V*_j_ to steady-state junctional conductance (*G*_j__ss_), measured at the end of the *V*_j_ step and normalized to the values recorded at ± 20 mV, is shown as the mean ± SEM of 4 pairs fit to a Boltzmann equation (dashed lines, Bii). The relationship demonstrates that the voltage-gating properties of Cx36 () or Cx36-EGFP (■) were almost identical with the exception of a slight shift of the transjunctional voltage required to elicit a conductance midway between *G*_j__max_ and *G*_j__min_ (Cx36 = 85 mV, Cx36-EGFP = 95 mV), consistent with the observation that changing the length of the carboxy-terminal tail alters the kinetics of *V*_j_ gating (Revilla et al., 1999). Scale = 50 μM.

A conspicuous feature of the Cx36-EGFP transgenic line is that brain sections prepared for GFP immunohistochemistry or epifluorescence contain discrete immunopositive or fluorescent puncta not seen in wildtype tissue. Although these puncta are widely distributed in the brain, their prevalence and/or ease of detection vary considerably with brain substructure. In particular, they are prominent in the retina (Feigenspan et al., 2004; Schubert et al., 2005) and in the glomerular and molecular layers of the olfactory bulb and cerebellum respectively (Fig. 1, but see also Fig. 2C for hippocampus and Fig. 3Aia for cortex). Their distribution, density, and ease of visualization suggest that the puncta represent dense aggregations of Cx36-EGFP at specialized subcellular structures. We previously exploited immunoelectron microscopy to demonstrate the presence of the Cx36-EGFP protein at gap junctions between dendrites located within the olfactory bulb glomerulus (Christie et al., 2005). As with the olfactory bulb (Figs. 1e,f), the presence of Cx36-EGFP molecules at gap junctions between dendrites in the cerebellum was demonstrated by immunoelectron microscopy (Figs. 1g,h). These findings are in accordance with the expectation that electrical synapses in the transgenic that are composed of Cx36 will also include Cx36-EGFP. Indeed, a comparison of Cx36-EGFP fluorescent clusters and Cx36-positive puncta, detected with an anti-Cx36 antibody, revealed that the vast majority of the latter colocalized with the former in the molecular layer of the cerebellum (421/455 Cx36 puncta also contained Cx36-EGFP, 92.5%; Fig. 2A).

We also examined the distribution of Cx36-EGFP puncta in the striatum and hippocampus (Figs. 2B and C respectively) in order to establish whether it conforms to previous characterization of Cx36 prevalence determined through use of anti-Cx36 antibodies. The density of Cx36 gap junctions linking the parvalbumin-positive cell network in the feline striatum was recently studied and observed to be enriched in the methionine–enkephalin-poor matrix (Fukuda, 2009). We attempted to reproduce those experiments in order to compare the distribution of Cx36-EGFP puncta in the mouse striatum but could not reveal differences in the distribution of methionine–enkephalin in the mouse striatal matrix, possibly due to species-specific differences in antigen distribution or antibody efficacy. However our measurements on the density of Cx36-EGFP puncta (*n* = 3 mice, mean ± SD = 21.26 ± 2.85 × 10^3^/mm^3^) are comparable to the previously reported values especially in light of the fact that Cx36 expression in the mouse striatum may not be restricted to parvalbumin-positive cells (Wellershaus et al., 2008). Furthermore, in accordance with that study (Fukuda, 2009) we observed an anterior–posterior gradient resulting in a significant 2.42-fold increase (SD = 0.9, *n* = 3 mice, *p* < 0.001) in the prevalence of Cx36-EGFP puncta in the posterior striatum (Fig. 2B). In the CA1 region of the hippocampus, parvalbumin-positive axo-axonic, bistratified and basket cells have their soma in the stratum pyramidale or adjacent stratum oriens, and a recent study in rat slices determined that all three interneuron classes form Cx36-immunopositive dendrodendritic gap junctions (Baude et al., 2007). In particular that study noted that Cx36 puncta was prominent in stratum radiatum towards stratum lacunosum-moleculare and in stratum lacunosum-moleculare and at the border between stratum oriens and alveus. An examination of the transgenic hippocampus similarly revealed the presence of parvalbumin and Cx36-EGFP coexpressing cells in the stratum pyramidale layer (Fig. 2Ci) with a dense accumulation of fluorescent puncta in the above-mentioned regions; in the stratum lacunosum-moleculare in particular (Fig. 2Cii).

These data in conjunction with previous studies of the Cx36-EGFP transgenic (Christie et al., 2005; Feigenspan et al., 2004; Schubert et al., 2005) and other investigations indicating that GFP puncta-rich regions in the transgenic brain are densely packed with Cx36 clusters in the wildtype (Fukuda et al., 2006; Meier et al., 2002; Rash et al., 2004) support the notion that GFP-containing puncta reflect the number and locations of electrical synapses composed of Cx36.

### The transgene does not restore electrical coupling to Cx36-deficient circuits in the knockout mouse

Immunohistochemical analysis indicated that EGFP-containing clusters were also present in the cerebral cortex (Fig. 3Aia) and that the vast majority of parvalbumin-positive cortical interneurons in the transgenic line also contained EGFP (98.98 ± 0.88%, *n* = 384 neurons, 3 mice; Fig. 3Aib,c). Therefore, we backcrossed the transgene into the Cx36 knockout genome and performed electrophysiological experiments (Fig. 3Aii) to determine if fast-spiking (FS) interneurons in layer II/III of the neocortex, shown previously to be parvalbuminergic and devoid of Cx36 function in the knockout (Blatow et al., 2003; Deans et al., 2001; Meyer et al., 2002), are electrically coupled in backcrossed progeny. These experiments would demonstrate whether the Cx36-EGFP protein expressed from the transgene can functionally compensate for the lack of genomic Cx36 expression. Contrary to our expectation, FS cells in slices derived from the compound genome Cx36^−/−^-TgCx36-EGFP were not coupled (0/11 pairs, *n* = 2) in comparison to their heterozygous littermates (Cx36^+/−^-TgCx36-EGFP; 15/23 pairs, CC ± SD = 0.018 ± 0.014, *n* = 7) or wildtype mice (Cx36^+/+^; 22/28 pairs, 0.025 ± 0.025, *n* = 7). The difference in CCs between the latter two genotypes was determined to not be significant (supplementary table; *p* = 0.34) although the probability of finding coupled cell pairs was somewhat higher for the wildtype (79% for Cx36^+/+^, 65% for Cx36^+/−^-TgCx36-EGFP).

The above data are consistent with the possibility that the Cx36-EGFP protein expressed from the transgene is functionally impaired and so we undertook to confirm this conclusion by examining its function in paired *Xenopus laevis* oocytes as done previously for other intercellular channel-forming molecules (Bruzzone et al., 2003). The experiments indicated that Cx36-EGFP consistently induced the assembly of intercellular channels that resulted in levels of conductance (*G*_j_) of the same order of magnitude (Fig. 3Bi; *G*_j_(μS) ± SEM = 4.8 ± 1.06, *n* = 18) as those measured in Cx36 injected pairs (2.98 ± 0.36, *n* = 21) whereas antisense-treated controls exhibited negligible coupling levels (0.04 ± 0.01, *n* = 16). To characterize the physiological behavior of channels composed of Cx36-EGFP, we also compared the voltage dependence of the two Cx36 protein variants. Junctional currents (*I*_j_) evoked by voltage steps of increasing amplitude indicated that, for both Cx36 and Cx36-EGFP, *I*_j_ decreased with time for a transjunctional voltage (*V*_j_) > + 40 mV. A plot of the normalized conductance (*G*_j_) versus *V*_j_ indicated that all parameters describing the voltage-gating behavior were almost identical for the two proteins thus demonstrating that addition of an EGFP tag to its carboxy-terminal tail did not appreciably alter the basic electrophysiological characteristics of Cx36 (Fig. 3Bii).

### Cx36-EGFP requires wildtype Cx36 for assembling into the electrical synapse

**Supplementary Fig. 1:**
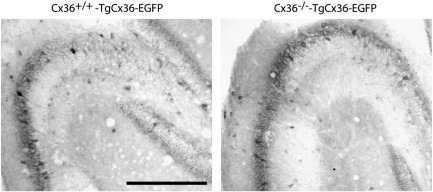
Comparable levels of Cx36-EGFP-expressing cells are present in transgenic mice regardless of genomic Cx36 expression. Immunohistochemistry of brain slices with anti-GFP antibody processed with the Vectastain ABC kit (Vector Laboratories) confirmed that numbers of Cx36-EGFP cells are comparable between homozygous wildtype (left; Cx36^+/+^-TgCx36-EGFP) and knockout (right; Cx36^−/−^-TgCx36-EGFP) Cx36 mice expressing the transgene indicating that the protein is synthesized in both genotypes. A portion of the hippocampus demonstrating the presence of the fusion protein in interneurons in the two genotypes is shown. Scale = 200 μM

**Fig. 4:**
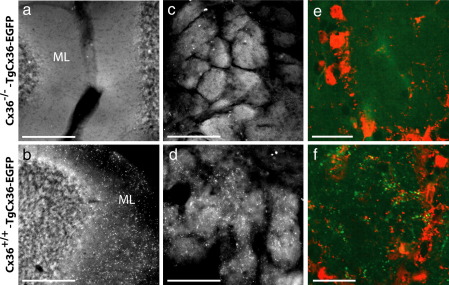
Cx36-EGFP puncta do not form in the absence of genomic Cx36 expression. The cerebellum (a, ML = molecular layer) and olfactory bulb glomeruli (c,e) of Cx36^−/−^-TgCx36-EGFP backcrossed progeny are devoid of GFP-containing puncta unlike the corresponding structures (b, cerebellum; d,f, olfactory bulb glomeruli) of their Cx36^+/+^-TgCx36-EGFP littermates. GFP puncta were observed both by epifluorescence (a,b,c,d) and immunohistochemical analysis (e,f) with anti-gfp and anti-calretinin antibodies (green and red respectively). The presence of puncta within glomeruli, demarcated by calretinin-positive cells, in Cx36^+/+^ mice (f) and absence in homozygous knockout littermates (e) is clearly noticeable at higher magnification. Scale: a,b,c,d = 100 μM, e,f = 25 μM.

The previous experiments indicated that interneurons of mice lacking Cx36 but containing the Cx36-EGFP protein expressed from the transgene were devoid of gap junction-mediated electrical communication. The ability of the transgenic protein to form functional intercellular channels in *Xenopus* oocytes similar to those composed solely of Cx36 however suggested that Cx36-EGFP was defective in a property unrelated to its conductance. To investigate the cause for the lack of electrical coupling further, we therefore examined the distribution of GFP-containing puncta in Cx36^−/−^-TgCx36-EGFP mice. As shown in Fig. 4, the olfactory bulb and cerebellum of transgenic mice lacking genomic Cx36 expression (a,c,e) were devoid of GFP puncta unlike the corresponding structures in their Cx36^+/+^-TgCx36-EGFP siblings (b,d,f). Immunohistochemical analysis with anti-GFP antibodies confirmed that the protein was still synthesized and present in the cell body of neurons (Supplementary Fig. 1) in Cx36 knockout animals expressing the transgene. These observations implicate a post-synthesis impediment, overcome by the coexpression of endogenous Cx36 protein, in assembling Cx36-EGFP into electrical synapses.

To explore the basis for this impediment as well as the relationship between Cx36 and Cx36-EGFP in coexpressing cells, we performed experiments in cultured HeLa cells expressing Cx36 and Cx36 chimeras tagged with fluorescent reporter proteins. HeLa cells were chosen for the ease with which multiple expression constructs may be introduced, the ability to clearly visualize junctional domains in adjacent pairs of connexin-expressing cells, and because they lack (Elfgang et al., 1995) or have infrequent and very low numbers of intercellular channels (Eckert et al., 1993); the cell type-specificity, prevalence, and magnitude of intercellular coupling in cultures prepared from embryonic or postnatal neurons, on the other hand, is unclear and may vary with culture duration (Arumugam et al., 2005).

**Fig. 5:**
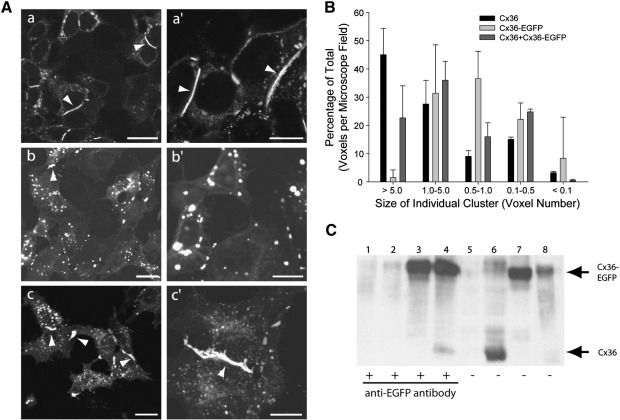
An obligatory requirement for Cx36 in the formation of Cx36-EGFP-containing junctional clusters in HeLa cells. A. The presence of connexin-containing clusters at the plasma membrane of HeLa cells expressing Cx36 (a,a′), or Cx36-EGFP (b,b′), or coexpressing the two (c,c′) was determined by immunocytochemistry with an anti-Cx36 antibody (a,a′) or by GFP epifluorescence (b,b′,c,c′). Arrowheads indicate the presence of large, clearly visible junctional clusters that are much more frequent in cells containing Cx36 than in cells expressing Cx36-EGFP alone. B. The percent contribution of individual connexin size classes formed within HeLa cells expressing Cx36 (■) or Cx36-EGFP () or Cx36 and Cx36-EGFP () to the total voxel count highlights the requirement of wildtype Cx36 for the formation of clusters in the largest size category. C. HeLa cells transfected with pRK5 (lanes 1,5), Cx36 (lanes 2,6), Cx36-EGFP (lanes 3,7), and co-transfected with Cx36 and Cx36-EGFP (lanes 4,8) were immunoprecipitated with an anti-EGFP antibody (lanes 1–4), blotted, and probed with an anti-Cx36 antibody. Arrows indicate the positions of the Cx36 and Cx36-EGFP proteins. Scale: a,b,c = 20 μM; a′,b′,c′ = 10 μM.

**Fig. 6:**
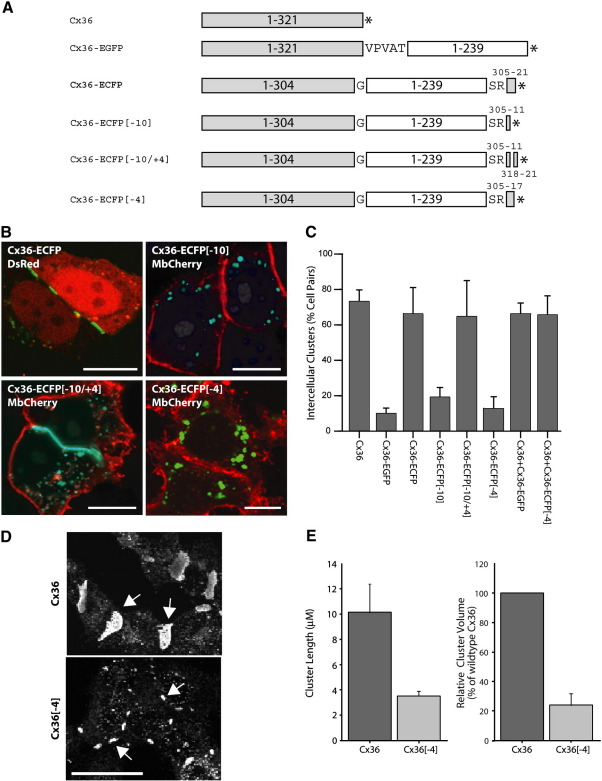
A carboxyl-terminal four amino acid motif is necessary for targeting Cx36 chimeras to the plasma membrane. A. Schematics depicting the unaltered and fluorescent protein-tagged variants of Cx36 transfected in HeLa cells. The Cx36 (gray box) and EGFP/ECFP (clear box) protein segments along with novel amino acid residues flanking the fluorescent protein segment inserted as a result of cloning are indicated. Also shown are the Cx36 residues retained in the construct after deleting specific carboxy terminal segments as well as the location of the translation termination codon (*). B. Cx36-ECFP and its derivatives bearing specific mutations at the carboxyl terminus were co-transfected into HeLa cells with either a cytoplasmic (DsRed) or membrane-bound (MbCherry) fluorescent reporter protein as indicated. The ability of the cyan fluorescent protein-tagged Cx36 variants to form protein complexes at the plasma membrane was determined by visual examination. Cx36-ECFP and Cx36-ECFP[− 10/+ 4] but not Cx36-ECFP[− 4] and Cx36-ECFP[− 10] were able to form large clusters at the membrane. C. The percentage of adjacent cell pairs expressing the specified Cx36 construct that contain intercellular clusters is plotted. The data highlight the significant handicap in the ability of Cx36-EGFP, Cx36-ECFP[− 4], and Cx36-ECFP[− 10] to form such aggregates, which, in the case of the first two, could be demonstrated to be overcome by coexpression with Cx36. D. The requirement of the terminal four amino acid residues for intercellular cluster formation is evident in cells expressing Cx36[− 4]. Such clusters (arrows indicate examples) are shorter (E, left) and less voluminous (E, right) than those formed by Cx36. Scale: B = 10 μM, D = 30 μM.

In the experiments displayed in Figs. 5 and 6, we identified the plasma membrane either by phase-contrast illumination or by surface exclusion/labeling with DsRed or MbCherry, an mcherry derivative (Shaner et al., 2004) of a membrane-bound GFP (De Paola et al., 2003). As shown in Fig. 5A, HeLa cells expressing Cx36 alone formed large deposits of membrane-bound protein between adjacent Cx36-expressing cells (Fig. 5Aa,a′) along with numerous smaller intracellular clusters. Typically, the larger aggregates formed along a substantial length of the shared plasma membrane (Fig. 5Aa′, arrowheads) and appeared similar to those reported for other dye transfer-promoting connexin clusters formed in HeLa cells (Falk, 2000). Cx36-EGFP formed smaller, spherical clusters within the cytoplasm (Fig. 5Ab,b′) and less frequently assembled surface clusters similar to those formed by Cx36 (arrowhead in Fig. 5Ab). In order to objectively characterize the apparent difference between connexin-containing clusters, we plotted the distribution of a defined number of clusters formed by the two constructs (*n* = 1288 Cx36; = 1328 Cx36-EGFP) into a set of size classes (Fig. 5B), noting that clusters located at the membrane between two connexin-expressing cells tended to be considerably larger than intracellular ones (see 5Aa′ or 5Ac′ for example). The percent contribution of clusters in each size category to the total measured voxel count was significantly different for the two (*χ*^2^ = 146.1, *df* = 4, *p* < 0.001) and demonstrated that Cx36 formed a greater number of large clusters (> 5 cluster size: Cx36, *n* = 34, mean ± SD = 11.93 ± 8.78; Cx36-EGFP, *n* = 7, 6.98 ± 1.72). The paucity of large junctional clusters in HeLa cells despite copious levels of Cx36-EGFP suggested that the protein was either not transported to sites of gap junction assembly or was not capable of being retained at those locations. A significant difference from wildtype in the contribution by each cluster category was evident even in cells in which the two Cx36 variants were co-transfected (Fig. 5Ac,c′; total cluster number = 1349, *χ*^2^ = 45.7, *df* = 4, *p* < 0.001), however a considerable improvement in the ability to form junctional clusters was evident in the increased number and size of clusters in the largest category (> 5 cluster size: *n* = 21, 9.71 ± 5.20). A comparison of the percent contribution of clusters in this size category to the total also confirmed the significant handicap in the ability of Cx36-EGFP to form membrane-localized clusters and the marked improvement when co-transfected with wildtype (Fig. 5B; mean ± SD, Cx36 = 45.1 ± 9.26; Cx36-EGFP = 1.55 ± 2.68; Cx36 + Cx36-EGFP = 22.66 ± 11.35; Cx36 vs Cx36-EGFP, *p* = 5.3 × 10^− 6^; Cx36 vs co-transfection, *p* = 5.4 × 10^− 3^; Cx36-EGFP vs co-transfection, *p* = 3.0 × 10^− 3^). The significant difference in cluster formation between cells transfected with wildtype alone and those co-transfected with the two Cx36 constructs may reflect variability in the relative amounts of the two proteins in individual cells and/or different intracellular fates for connexons composed of varying amounts of the two proteins.

In order to determine whether connexin complexes in coexpressing cells contained both proteins, lysates were immunoprecipitated with an anti-EGFP antibody and probed with an anti-Cx36 antibody. As shown in Fig. 5C, Cx36 could be specifically immunoprecipitated from complexes containing Cx36-EGFP (lane 4) — the procedure did not precipitate Cx36 from Cx36-expressing cells lacking Cx36-EGFP (lane 2) although a substantial amount of the protein was present in that lysate (lane 6). These experiments suggest that the Cx36 and Cx36-EGFP proteins participate in a common complex. One potential explanation of these findings is that the ability of Cx36 to cause incorporation of Cx36-EGFP into plasma membrane connexin clusters is a consequence of heteromerization in the same connexon molecule.

### A carboxy terminal domain is necessary for incorporating Cx36 into surface complexes

**Supplementary Fig. 2:**
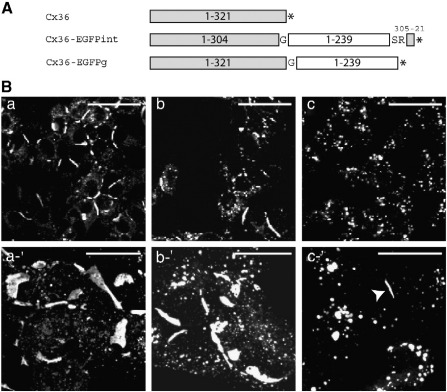
Formation of intercellular clusters by Cx36 variants. A. Schematics illustrating the wildtype Cx36 and two variants of Cx36-EGFP (Cx36-EGFPg and Cx36-EGFPint). The protein coding fragments of Cx36 and EGFP (gray and clear rectangles respectively) along with amino acid residues flanking the latter and location of the translation termination codon (asterisk) are indicated. The ability of the two EGFP fusion constructs to form intercellular clusters typically seen with Cx36 (a, a′) is illustrated in B. The images were obtained after immunocytochemical visualization with an anti-Cx36 (Chemicon) antibody and represent maximum projections of optical sections acquired on a Lecia SP5 confocal microscope. The results show that Cx36-EGFPint frequently forms large aggregates of the protein at intercellular domains between adjacent Cx36-EGFPint-expressing cells (b, b′), whereas Cx36-EGFPg forms smaller dense clusters within the cytoplasm (c, c′) and only occasionally forms intercellular aggregates reminiscent of those formed by Cx36 (arrowhead in c′). Scale bar, upper panels = 50 μM; lower panels = 20 μM.

The preceding experiments suggest that a defect in Cx36-EGFP prevents it from being incorporated into gap junction complexes. This defect can at least partially be rescued by the coexpression of the wildtype, unaltered form of the protein. One possibility is that the EGFP molecule disallows access to a peptide domain recognized by relevant chaperones and/or assembling molecules. Such protein–protein interactions have been determined to be important in assembling complexes at the synapse (Kim and Sheng, 2004). Indeed, the conserved carboxy terminus of Cx36 has a consensus motif of a typical ligand for a type 2 PDZ domain (Hung and Sheng, 2002) and has been shown to interact with the ZO-1 protein via this sequence (Li et al., 2004a). We therefore generated a construct in which an enhanced cyan fluorescent protein (ECFP) was inserted within the intracytoplasmic carboxy-terminal tail of Cx36 (Cx36-ECFP) 15 amino acid residues from the terminus (Fig. 6A). Unlike Cx36-EGFP, the ECFP-tagged version was able to form typical, large, membrane-localized clusters that abutted the common boundary of adjacent connexin-expressing HeLa cells demonstrating that the fluorescent reporter tag does not intrinsically inhibit connexin molecules from trafficking to and incorporating in gap junctions (Fig. 6B). Further deletions from the carboxy terminus of this Cx36 tagged variant identified the final four residues as being necessary for the ability of Cx36-ECFP to form large clusters (Figs. 6A,B). Thus constructs lacking the final 10 and final 4 amino acids (Cx36-ECFP[− 10] and Cx36-ECFP[− 4] respectively) were unable to form them whereas one containing the last 4 but lacking the preceding 6 (Cx36-ECFP[− 10/+ 4]) was able to do so. Results pertaining to the frequency of intercellular cluster formation between adjacent pairs of connexin-expressing cells are shown in Fig. 6C. The data demonstrate that Cx36-EGFP (% of clusters, mean ± SD = 10.07 ± 2.94,), Cx36-ECFP[− 10](19.31 ± 5.32), and Cx36-ECFP[− 4] (12.73 ± 6.72) are significantly impaired in their ability to incorporate the tagged versions of Cx36 into the membrane compared to the untagged, wildtype form of the protein (73.32 ± 6.41; *p* < 0.01). Two additional constructs demonstrated that the difference in cluster formation exhibited by Cx36-ECFP and Cx36-EGFP could not be attributed to differences in peptide sequences between the two. The first contained a replacement of ECFP in Cx36-ECFP with EGFP thus inserting EGFP within the cytoplasmic carboxy terminus of Cx36 (Cx36-EGFPint), and the second replaced the ‘VPVAT’ peptide sequence between the Cx36 and EGFP domains in Cx36-EGFP with a glycine residue as in Cx36-ECFP (Cx36-EGFPg). Whereas the former formed clusters similar to those formed by Cx36-ECFP, the latter behaved like Cx36-EGFP (Supplementary Fig. 2).

To further demonstrate the importance of the terminal four ‘SAYV’ residues of Cx36 in gap junction formation, we evaluated the ability of Cx36 lacking the motif (Cx36[− 4]) to assemble into membrane-localized clusters in HeLa cells. These experiments would indicate whether the importance of the motif is restricted to the fluorescent protein-tagged variants of Cx36 or whether it extends to the wildtype Cx36 molecule as well. Protein aggregates composed of Cx36[− 4] did not form the prominent intracellular clusters characteristic of their fluorescent-tagged counterparts but a deficiency in intercellular cluster formation in cells transfected with this construct was nevertheless evident — clusters appeared smaller with multiple clusters frequently lining the membrane between two Cx36[− 4]-expressing cells (Fig. 6D). This deficit was quantified by comparing the length and volume of intercellular clusters formed by Cx36[− 4] and Cx36 (Fig. 6E). Cx36[− 4] clusters were determined to be significantly shorter in length (Cx36: *n* = 460, mean ± SD = 9.30 ± 6.55 μM; Cx36[− 4]: *n* = 397, 3.57 ± 3.01 μM; *p* = 0.007) and less voluminous (Cx36: *n* = 216, mean voxel number ± SD = 626.84 ± 223.83; Cx36[− 4]: *n* = 216, 147.16 ± 60.70, *p* = 0.024; mean voxel number of Cx36[− 4] clusters = 23.85 ± 7.82% of Cx36 clusters). The limited ability of Cx36[− 4] to form intercellular clusters was also evident when we compared the length of the 60 largest gap junctions (20 per experiment) formed by either Cx36[− 4] or the wildtype protein (Cx36: 21.87 ± 1.11 μM; Cx36[− 4]: 8.97 ± 2.36 μM; *p* = 0.001). These analyses confirm the importance of the motif for assembling Cx36 into intercellular clusters.

## Discussion

In the experiments described above, we have characterized a Cx36 transgenic protein, examined the defective targeting of the protein in transgenic neural tissue and transfected HeLa cells, and thus determined an obligatory requirement for a carboxy terminal motif in incorporating Cx36 into electrical synapses. Although previous studies identified specific protein associations mediated by the same motif in non-neural cell lines, our experiments indicate that its availability is vital for the formation of Cx36-containing electrical synapses *in vivo*.

The process in which the Cx36-EGFP molecule is deficient is unclear. Microscopic examination of Cx36-EGFP clusters in the compound transgenic (Cx36^−/−^-TgCx36-EGFP) reveals it to be lacking at the locations one would normally expect to find gap junctions — its absence was functionally confirmed by the failure of the transgene to couple parvalbumin-positive fast-spiking cells in mice with a Cx36KO genotype. This localization defect of the Cx36-EGFP molecule was also exhibited in HeLa cells and could be the result of either the inability of the chimeric protein to be directed to gap junction assembly sites or, alternatively, of its failure to be retained at those sites. The similarity in the appearance and distribution of clusters formed by Cx36-ECFP[− 10] and Cx36-ECFP[− 4] to those formed by Cx36-EGFP (viz., mainly intracellular) suggests that the defect in all three constructs is the same. Together, these observations point to a direct involvement of the terminal four amino acid residues in assembling Cx36 into gap junctions, which was also evident in our demonstration that gap junctions formed by Cx36[− 4] were significantly smaller than those formed by Cx36. Our observations imply that the primary reason for the inability of Cx36-EGFP to form gap junctions between HeLa cells and neurons is inaccessibility to the carboxy terminal motif owing to the presence of EGFP. That the deficiency is overcome by the presence of wildtype unaltered Cx36 in both, HeLa cells and transgenic neural tissue, indicates it to be a recessive property capable of trans complementation. Thus, coexpression of the transgene and genomic Cx36 results in Cx36-EGFP incorporation into functional electrical synapses that electrically couple parvalbumin-positive fast-spiking cortical circuits. The most likely mechanistic explanation for this complementation, supported by immunoprecipitation experiments indicating the presence of complexes containing both proteins, is their heteromerization into connexons generated by coexpressing cells. In this scenario, one or more unaltered Cx36 subunits with an intact carboxy terminus are sufficient to allow such heteromeric connexons to interact with cellular molecules involved in gap junction trafficking and assembly.

The defect in the formation of clusters at distributed junctional zones and its rescue by wildtype Cx36 in both, HeLa cells and neurons, also suggests that the underlying impediment is the same in both cell types. Thus our studies seem to indicate that the terminal four amino acid residues identified by deletion mutagenesis as being important for the presence of the tagged Cx36 at the plasma membrane regulates a general, cell-independent property of assembly rather than a neuron-specific one. Protein co-factors that may interact with this motif to direct the intracellular fate of Cx36-EGFP bearing connexons are thus expected to be present in a variety of cells. The formation of intercellular channels with Cx36-like function composed solely of Cx36-EGFP in *X. laevis* oocytes confirms that the protein is able to contribute to electrical transmission when it is a constituent of the channel and suggests that it is processed and trafficked normally in these cells. While the latter suggestion could imply that a different mechanism for intercellular channel formation operates in this system, it is also possible that the considerable overexpression resulting from RNA injection or that the lower temperatures at which Cx36-EGFP-expressing oocytes were incubated may promote the surface delivery of connexons. The increase in protein trafficking to the cell surface at lower temperatures has been reported previously (Wang et al., 2009) and the electrophysiological characterization of α7 nicotinic acetylcholine receptors was notable for its dependence on the expression system used (Millar, 2008). While the latter studies appeared to support the existence of different mechanisms for the surface targeting and function of the receptor, the subsequent demonstration that functional expression was correlated with the presence of the chaperone, Ric-3, and could be restored by its coexpression in chaperone-lacking cells suggested that a single mechanism operates across different cell types. Our experiments show that Cx36-EGFP (and see Zoidl et al., 2002) and a Cx36 mutant protein lacking the ‘SAYV’ motif are able to assemble lower levels of intercellular clusters in HeLa cells even in the absence of complementation, suggesting either that some gap junction formation can occur even in the absence of carboxy terminal access or that an alternative less efficient mechanism operates simultaneously. The absence of puncta and electrical coupling *in vivo* in Cx36KO mice expressing the transgenic may be interpreted as evidence for a more restrictive requirement for gap junction formation in neurons although the presence of small clusters of intercellular channels composed of Cx36-EGFP that are unable to provide electrical coupling cannot be ruled out. An elegant series of experiments has previously demonstrated that the relationship between channel number and junctional conductance is complex and that coupling is often lacking if cell pairs contain small intercellular channel clusters (Bukauskas et al., 2000). The simplest hypothesis consistent with all our observations invokes the interaction of another factor with the carboxy terminal motif as a prerequisite for gap junction formation. However it is also possible that different mechanisms may operate in diverse experimental systems.

Previous biochemical and immunological evidence have demonstrated an interaction between Cx36/Cx35 and the scaffold protein ZO-1, which is expressed in both HeLa cells and neurons, and experiments have even identified the carboxy terminal residues as being responsible for mediating this interaction (Ciolofan et al., 2006; Flores et al., 2008; Li et al., 2004a,b; Rash et al., 2004). However, we note that earlier immunohistochemical characterization of retina and other brain regions has revealed the existence of abundant Cx36 puncta, some of which lack the ZO-1 protein (Li et al., 2004a; Rash et al., 2004). This suggests that other proteins – possibly other scaffold proteins (Ciolofan et al., 2006; Puller et al., 2009) – may also subserve the same function and that the defect exhibited by Cx36-EGFP may be independent of its ability to interact with ZO-1. Although our experiments do not identify the responsible chaperones they do suggest that this interaction is obligatory for the incorporation of Cx36 and the subsequent formation of a functional electrical synapse.

Fluorescent protein-tagged connexins have found extensive use in the cell biological and functional characterization of gap junction constituent molecules. An earlier study had also examined cluster formation and function of a GFP-tagged Cx36 molecule similar to the one we describe (Zoidl et al., 2002). Unlike the previous study, however, we compare the subcellular distribution of Cx36-EGFP with that of wildtype Cx36 applying a detailed statistical evaluation of cluster distribution and methods to discriminate between intracellular and membrane-bound clusters in large cell populations. We note that their conclusion that a Cx36 molecule lacking the carboxy terminus has an unaltered subcellular distribution contradicts our findings as well as those reported by Li et al. (2004a). This discrepancy highlights the difficulty in investigating the anatomical and functional properties of neuronal gap junctions *in vitro*, emphasizing the importance of *in vivo* characterization of modified gap junction components. Another HeLa cell study of a Cx43-EGFP chimera similar to Cx36-EGFP is also worth considering (Hunter et al., 2005) in light of our results. The Cx43-EGFP protein was observed to be different from wildtype Cx43 in that it formed larger-than-wildtype clusters at the plasma membrane. Subsequent analysis led the authors to conclude that the inability of ZO-1 to interact with the carboxy terminal sequences of Cx43 in the chimera resulted in unregulated growth of the gap junction. The Cx36-EGFP, Cx36-ECFP[− 10] and Cx36-ECFP[− 4] protein behave differently — they have difficulty forming clusters at the plasma membrane. The differences between our observations and those reported for Cx43 highlight intrinsic differences in the trafficking and assembly of the two connexins, possibly those mediated by the carboxy terminus. However, the similarities in the two constructs regarding the impact of the tag on gap junction morphology emphasize that careful attention be paid to the site of insertion of fluorescent reporter tags in the study of connexins.

Determining the contribution of electrical synapses to neural activity is facilitated by knowledge of their architecture within electrically-coupled circuits. Recent findings have indicated their organization to be very complex (Fukuda, 2007, 2009; Hestrin and Galarreta, 2005); a consequence of specificity in expression, in the cell-types that participate in their formation, and in their anatomical localization. For example, Cx36 within the mouse main olfactory bulb is expressed by mitral/tufted cells, and specific cells in the periglomerular and granule cell regions (Kosaka et al., 2005), and electrical synapses formed by mitral cells are located primarily within the glomerulus resulting in the formation of glomerular-specific mitral cell assemblies (Christie et al., 2005). This specific distribution of gap junction-forming molecules may be critical for processing odor-related information. Procedures to identify electrical synapse-expressing cells and sites have, however, numerous problems or are cumbersome and/or restricted to fixed brain tissue. Antibodies used to identify the location of Cx36 protein clusters in brain tissue are often non-specific (Meier et al., 2002) or result in high background (Li et al., 2004a), and reporter mice frequently exhibit misexpression or loss-of-expression of the reporter protein (Deans et al., 2002; Feigenspan et al., 2004; Wellershaus et al., 2008). The requirement of the wildtype protein for incorporating Cx36-EGFP into the gap junction in the Cx36-EGFP transgenic line has the obvious benefit that only “genuine” electrical synapses will contain the fluorescent reporter enabling their easy identification and quantification. Thus the Cx36-EGFP transgenic presents a considerable advantage over other antibody- or electron microscopy-based methods (Fukuda et al., 2006) in the attempt to study Cx36 electrical synapse distribution, particularly in conjunction with techniques used in living tissue.

## Experimental methods

All experiments were performed in accordance with institutional guidelines. Experimental procedures in Dundee were authorized by a U.K. Home Office project licence (PPL 60/3533).

### Imaging GFP in transgenic brain slices

Adult Cx36-EGFP mice between 2 and 6 months of age were deeply anesthetized with ketamine-xylasine and perfused transcardially with 4% paraformaldehyde (PFA)/phosphate-buffered saline (pH 7.4) after which 50 µM sagittal sections were prepared from the brain. GFP was imaged directly by epifluorescence illumination using an upright microscope (BX51 Olympus) or a Leica TCS SP2 confocal microscope. Alternatively free floating slices were immunolabeled with rabbit anti-GFP (Zymed 1:5000) or double immunolabelled with the following primary, rabbit anti-GFP and mouse anti-calbindin D28K (Swant, 1:2000) or rabbit anti-GFP and mouse anti-Calretinin (Swant, 1:5000) or rabbit anti-GFP and mouse anti-parvalbumin (Sigma, 1:1000), and secondary, Alexa-488-conjugated goat anti-rabbit (1:2000) and Alexa-555-conjugated goat anti-mouse (1:2000) antibody combinations. Immunolabelled images were acquired by sequential scanning of the desired regions with a Leica TCS SP2/5 confocal microscope. The colocalization of Cx36-EGFP with Cx36 immunopositive puncta in the cerebellar molecular layer was demonstrated via immunohistochemistry with mouse anti-Cx36 (Chemicon 1:2000) and Alexa-555-conjugated goat anti-mouse (1:2000) primary and secondary antibodies respectively. Quantitation was performed on images obtained from 4 different mice.

Quantification of Cx36-EGFP puncta in the striatum was done after acquiring a confocal stack at 0.2 μM intervals and assembling as a 3D projection in MetaMorph (Molecular Devices). EGFP-containing puncta were quantified from multiple slices containing different regions of the striatum and for three transgenic animals. The anterior–posterior distribution of Cx36-EGFP fluorescent puncta (Fig. 2B) was determined on slices from 3 brains cut coronally. A single confocal image (357 × 357 μM) from the centre of the striatal field was obtained at identical laser and acquisition settings for the first six (anterior) and last six (posterior) slices determined visually to contain the striatum. These 24 images (two images per slice, one from each hemisphere) were processed identically in metamorph to highlight puncta, whose number were then noted and recorded in the box plot as the median, 10th, 25th, 75th and 90th percentiles for the anterior and posterior striatal regions of each mouse. It is estimated that the data reflect the prevalence of Cx36-EGFP puncta within the proximal and distal 500 μM of mouse striatum.

To demonstrate the coexpression of Cx36-EGFP and parvalbumin in cortex, we performed immunohistochemistry on Cx36-EGFP brain slices sequentially with mouse anti-parvalbumin antibody (Sigma, 1:1000) first, detected with Alex-555 anti-mouse secondary, followed by immunolabelling with anti-GFP, detected with DAB immunochemistry (Vectastain kit, Vector laboratories). Consecutive fluorescence and brightfield images were taken to detect parvalbumin- and Cx36-EGFP-expressing neurons respectively. The total number of parvalbumin neurons in the cortex that also contained GFP was determined for three transgenics and expressed as a percentage.

### Electron microscopy

Anesthetized adult mice were perfused transcardially first with heparin-containing saline followed by PFA fixative solutions at variable pH (4% PFA in 2% sodium acetate adjusted to pH 6.5, immediately followed with 4% PFA/0.05% glutaraldehyde in 0.1 M sodium carbonate–sodium bicarbonate buffer, pH 11). After perfusion the olfactory bulbs and cerebella were dissected and postfixed in the second PFA solution without glutaraldehyde for 2–3 h at RT. Vibratome sections of 50 µM thickness were cut, permeabilized, and incubated with rabbit anti-GFP. Preembedding immunogold labeling was performed, using Ultra Small gold conjugated single Fab goat anti-rabbit antibody (Aurion, 1:100 dilution). Gold particles were silver intensified using the enhancement kit as recommended by the manufacturer (R-GENT SE-EM, Aurion). Sections were then treated with osmium tetroxide, dehydrated, flat embedded in Epon and cured at 70 °C for 24 h. The areas of interest were cut out from the flat embedded tissue and re-embedded onto Epon blocks. Ultrathin (60–70 nm) sections were cut from the blocks, placed on Pioloform-coated cooper single-slot grids, contrasted with uranil acetate and lead citrate and examined with a Zeiss 10 electron microscope.

### Electrophysiology

Sagittal neocortical brain slices of 250 μM thickness were prepared from 14 to 21 day-old C57Bl6 (wildtype), and Cx36^+/−^ and Cx36^−/−^ mice carrying the Cx36-EGFP transgene (Cx36^+/−^-TgCx36-EGFP and Cx36^−/−^-TgCx36-EGFP respectively). Brain slice preparation, visualization of neurons in living slices, and description of extra- and intracellular solutions are described elsewhere (Blatow et al., 2003). Recordings were obtained at 33 °C in current clamp from pairs of neurons < 100 μM apart in layer II/III with an appearance and firing pattern typical of fast-spiking cells (Blatow et al., 2003). Gap junction coupling was assessed in the presence of inhibitors of AMPA/kainate (CNQX, 20 μM) and GABA_A_ (SR-95531, 15 μM) receptors by repetitive injection of hyperpolarizing pulses (− 500 or −1000 pA, 700 ms duration, 30 sweeps) alternating to both cells. After averaging, coupling coefficient (CC; supplementary table) was determined as the ratio between the amplitude of the voltage deflection evoked in the noninjected cell to the response evoked in the injected cell measured at the end of the pulse. Cells were considered coupled only if both cells had a coupling coefficient greater than 0.001 and exhibited a postsynaptic response with a clearly recognizable shape, i.e. the postsynaptic response was a negative voltage deflection whose onset and termination coincided with the injected current in the presynaptic cell.

For experiments in *Xenopus* oocytes, Cx36 and the tagged constructs subcloned in pRK5 were linearized with Hind III, gel purified and used as template (1 µg DNA) to produce capped RNA using the mMessage mMachine kit (Ambion). *In vitro* transcription, preparation of *Xenopus* oocytes, injection of antisense oligonucleotides (3 ng/cell, to suppress activity of the endogenous *Xenopus* Cx38 (XenCx38)) or a mixture of antisense plus the specified RNA (10–40 ng/cell), and electrical recordings were performed as described elsewhere (Bruzzone et al., 1994).

For measurements of junctional conductance, both cells of a pair were initially clamped at − 40 mV and alternating pulses of ± 10–20 mV were delivered to one cell. The current recorded in the cell clamped at − 40 mV was divided by voltage to yield conductance (*G*_j_). To determine voltage-gating properties, voltage in one cell was altered in 20 mV steps (over a range of ± 120 mV) while clamping the second cell at − 40 mV. Currents were measured 5 s after the onset of the voltage pulse, at which time they approached steady state (*I*_jss_), and the macroscopic conductance (*G*_j__ss_) was calculated by dividing *I*_jss_ by *V*_j_. *G*_j__ss_, normalized to the values determined at ± 20 mV, was plotted against *V*_j_. Data describing their relationship were fit to a Boltzmann relation of the form: *G*_j__ss_ = {(*G*_j__max_ − *G*_j__min_)/(1 + exp[A(*V*_j_ − *V*_0_)])} + *G*_j__min_, where *G*_j__max_ (set as unity) is the maximum conductance, *G*_j__min_ is the residual conductance at large values of *V*_j_, and *V*_0_ is the transjunctional voltage at which *G*_j__ss_ = (*G*_j__max_ − *G*_j__min_)/2. The constant *A* (= *nq*/*kT*) represents the voltage sensitivity in terms of gating charge as the equivalent number (*n*) of electron charges (*q*) moving through the membrane, *k* is the Boltzmann constant, and *T* is the absolute temperature.

### Plasmids

EGFP, ECFP, mCherry and DsRED plasmids were obtained from Clontech (USA). The Cx36 construct used to transfect HeLa cells consisted of the Cx36 open reading frame cloned in pRK5. All other constructs (Fig. 6A) were variants of this plasmid prepared by PCR amplification and subcloning of the relevant segments. Cx36-EGFP recreated the tagged Cx36 protein expressed in the transgenic line whereas Cx36-ECFP was generated by inserting an ECFP fragment within the cytoplasmic carboxy terminal domain of the Cx36 protein. Alterations to Cx36-ECFP resulted in the specified carboxy terminal deletions. All constructs were sequenced and determined to encode the proteins illustrated in Fig. 6A. MbCherry was generated by replacing the GFP fragment in mGFP (De Paola et al., 2003) with mCherry (Shaner et al., 2004).

### Cell culture, transfection, and immunocytochemistry

HeLa cells were transiently transfected (20 µg total plasmid/10 cm dish or 24 µg/24-well dish) using the calcium-phosphate precipitation method. Coexpression of connexin constructs was accomplished by transfecting a plasmid mixture comprising a 5:1 molar excess of Cx36 to the Cx36-tag (typically 0.75 μg Cx36/0.25 μg Cx36-EGFP). Control transfections for immunocytochemistry and Western blotting were performed using vector DNA (pRK5). For microscopic examination of transfected cells, HeLa cells were plated on Poly-d-lysine coated cover slips. Immunocytochemistry was always performed for cells transfected with Cx36 alone with mouse anti-Cx36 (Chemicon 1:2000) and Alexa-555-conjugated goat anti-mouse (1:2000) primary and secondary antibodies respectively. Tagged constructs were often immunostained with the anti-Cx36 antibody but more frequently visualized on the basis of GFP epifluorescence and, when co-transfected with Cx36, were immunostained with rabbit anti-GFP antibody (Alexa-488-conjugated anti-rabbit secondary) or visualized for GFP fluorescence. The appearance and frequency of inter- and intracellular GFP clusters did not seem to differ with the procedure used for visualization.

### Quantification of HeLa cell clusters

Images were acquired on a Leica TCS SP5 confocal microscope under sequential scan settings appropriate for the antibody conjugated fluorophores and fluorescence reporter proteins. Optical sections for cells in the field of view (with a step size of 0.4–0.6 μM) were acquired and processed in MetaMorph or LAS-AF (Leica Microsystems). Number of cell pairs with intercellular clusters (Fig. 6C) was determined by examining each optical section for the presence of a distinct cluster, regardless of size, between two adjacent connexin-expressing cells. In this case, the cytoplasm/plasma membrane boundary was determined by phase-contrast illumination or by localization of the co-transfected cytosolic (DsRed) or membrane bound (MbCherry) reporter construct. This determination was done for a total of three transfection experiments per construct, each experiment comprising a minimum of 25 cell pairs.

For a comparison of the clusters formed by Cx36 and Cx36-EGFP, cluster size was determined in MetaMorph by combining the optical sections of a field of view consisting of multiply transfected cells into a single 3-D image. This was then thresholded to generate a minimum of 120 objects. In order to overcome differences in laser settings used to detect EGFP, ECFP, and Alexa fluorescence, the percentage contribution of the voxel number/size of each object to the combined voxel size of a maximum of 150 thresholded objects in each field of view was determined. This was measured for nine such fields (three different transfections, three fields each) and the relative voxel/cluster size was classified into the different size groups shown in Fig. 5B. The plot illustrates the percent contribution of the total of all clusters in each size group to the combined voxel size of all thresholded clusters averaged for each transfection.

The skeletal length and voxel size of intercellular clusters formed by Cx36 and Cx36[− 4] (Fig. 6E) was determined from a total of three transfection experiments, each comprising three independent fields of view. After immunolabelling with anti-Cx36 (Chemicon 1:2000) and Alexa-555-conjugated goat anti-mouse (1:2000) primary and secondary antibodies respectively, images were obtained at optical sections 0.2 μM apart at identical laser and acquisition settings. The maximum skeletal length was measured for all clusters that were visually identified to lie at the plasma membrane in a maximum projection of the optical sections using LAS-AF software. Cluster volume was measured in MetaMorph as described above and determined for an equal number of the largest intercellular clusters formed by the two constructs identified at identical threshold settings (25, 25 and 22 clusters per field of view for each of the three experiments respectively). The latter data are plotted as the mean cluster volume formed by Cx36[− 4] relative to those formed by wildtype Cx36.

### Immunoprecipitation and Western blotting

Transfected HeLa cells were briefly sonicated in 20 mM Tris–HCl (pH 8.0) containing 150 mM NaCl, 1% Triton-X-100, 10% glycerol, 1 mM DTT, 1 mM EGTA, 1.5 mM MgCl_2_ and protease inhibitors, and the supernatants were extracted by centrifugation at 13,000 × *g* for 10 min. 200–250 μg of protein extracts were incubated with mouse anti-GFP antibody (Roche Applied Science) and precipitated complexes were captured by Protein A/G agarose beads (Santa Cruz Biotechnology), immobilized to polyvinylidene difluoride (PVDF) membranes, and incubated with rabbit anti-Cx36 (Zymed). For comparison, 1/10th of the non-immunoprecipitated protein lysate was also loaded on the gel and subjected to Western blotting. Horseradish peroxidase-conjugated secondary antibody was used in a non-radioactive procedure (ECL, Amersham Biosciences) for detection.

## Appendix A Supplementary data

Supplementary table Coupling coefficients exhibited by fast-spiking interneurons in the neocortex*.
