# Supplementary material for: In vivo evidence for the involvement of the carboxy terminal domain in assembling connexin 36 at the electrical synapse
Source: Mol Cell Neurosci. 2010 Sep;45(1-3):47–58. doi: 10.1016/j.mcn.2010.05.008 (PMC3025355; doi:10.1016/j.mcn.2010.05.008)
Supplement: Supplementary table — Coupling coefficients exhibited by fast-spiking interneurons in the neocortex*. [file mmc1.doc]

# Supplementary Table:

Coupling Coefficients exhibited by fast-spiking interneurons in the neocortex*

|  | Cx36^+/+^ | Cx36^+/-^-TgCx36-EGFP |
| --- | --- | --- |
|  |  |  |
|  | 0.030 | 0.002 |
|  | 0.010 | 0.009 |
|  | 0.032 | 0.010 |
|  | 0.051 | 0.029 |
|  | 0.024 | 0.041 |
|  | 0.004 | 0.014 |
|  | 0.012 | 0.005 |
|  | 0.019 | 0.009 |
|  | 0.032 | 0.045 |
|  | 0.099 | 0.002 |
|  | 0.001 | 0.031 |
|  | 0.012 | 0.018 |
|  | 0.001 | 0.014 |
|  | 0.083 | 0.004 |
|  | 0.003 | 0.030 |
|  | 0.035 |  |
|  | 0.029 |  |
|  | 0.024 |  |
|  | 0.018 |  |
|  | 0.013 |  |
|  | 0.005 |  |
|  | 0.005 |  |
| Average | 0.025 | 0.018 |
| Standard Deviation | 0.025 | 0.014 |
|  |  |  |

*1 CC per cell pair displayed.
